# Safety and Pharmacokinetics of Casirivimab and Imdevimab (CAS + IMD) in Pediatric Outpatients With COVID-19

**DOI:** 10.1093/jpids/piae105

**Published:** 2024-10-01

**Authors:** Thomas D Norton, Mazhar Thakur, Samit Ganguly, Shazia Ali, Jesse Chao, Alpana Waldron, Jing Xiao, Kenneth C Turner, John D Davis, Susan C Irvin, Cynthia Pan, Dominique Atmodjo, Andrea T Hooper, Jennifer D Hamilton, Mohamed Hussein, Danise Subramaniam, Lilia Roque-Guerrero, Anita Kohli, Eleftherios Mylonakis, Gregory P Geba, Edward Cox, Ned Braunstein, Paula Dakin, Bari Kowal, Rafia Bhore, A Thomas DiCioccio, Diana Hughes, Gary A Herman, for the Trial Investigators

**Affiliations:** Regeneron Pharmaceuticals, Inc., Tarrytown, New York, USA; Regeneron Pharmaceuticals, Inc., Tarrytown, New York, USA; Regeneron Pharmaceuticals, Inc., Tarrytown, New York, USA; Regeneron Pharmaceuticals, Inc., Tarrytown, New York, USA; Regeneron Pharmaceuticals, Inc., Tarrytown, New York, USA; Regeneron Pharmaceuticals, Inc., Tarrytown, New York, USA; Regeneron Pharmaceuticals, Inc., Tarrytown, New York, USA; Regeneron Pharmaceuticals, Inc., Tarrytown, New York, USA; Regeneron Pharmaceuticals, Inc., Tarrytown, New York, USA; Regeneron Pharmaceuticals, Inc., Tarrytown, New York, USA; Regeneron Pharmaceuticals, Inc., Tarrytown, New York, USA; Regeneron Pharmaceuticals, Inc., Tarrytown, New York, USA; Regeneron Pharmaceuticals, Inc., Tarrytown, New York, USA; Regeneron Pharmaceuticals, Inc., Tarrytown, New York, USA; Regeneron Pharmaceuticals, Inc., Tarrytown, New York, USA; Regeneron Pharmaceuticals, Inc., Tarrytown, New York, USA; Bio-Medical Research, Miami, Florida, USA; Arizona Liver Health, Tucson, Arizona, USA; Infectious Diseases Division, Warren Alpert Medical School of Brown University, Providence, Rhode Island, USA; Department of Medicine, Houston Methodist Hospital, Houston, Texas, USA; Regeneron Pharmaceuticals, Inc., Tarrytown, New York, USA; Regeneron Pharmaceuticals, Inc., Tarrytown, New York, USA; Regeneron Pharmaceuticals, Inc., Tarrytown, New York, USA; Regeneron Pharmaceuticals, Inc., Tarrytown, New York, USA; Regeneron Pharmaceuticals, Inc., Tarrytown, New York, USA; Regeneron Pharmaceuticals, Inc., Tarrytown, New York, USA; Regeneron Pharmaceuticals, Inc., Tarrytown, New York, USA; Regeneron Pharmaceuticals, Inc., Tarrytown, New York, USA; Regeneron Pharmaceuticals, Inc., Tarrytown, New York, USA

**Keywords:** COVID-19, monoclonal antibodies, outpatients, pediatrics, treatment

## Abstract

The safety of casirivimab + imdevimab (CAS + IMD) (anti-severe acute respiratory syndrome coronavirus 2 [SARS-CoV-2] monoclonal antibodies [mAbs]) in pediatric outpatients with coronavirus disease 2019 (COVID-19) was evaluated in a randomized phase 1/2/3 trial. Consistent with adults, CAS + IMD was generally well tolerated with low drug-induced immunogenicity rates. The findings support the development of next-generation anti-SARS-CoV-2 mAbs for at-risk pediatric patients.

## Teaser

Casirivimab and imdevimab (CAS + IMD) treatment was well-tolerated in pediatric outpatients with ≥1 risk factor for severe coronavirus disease 2019 (COVID-19), with similar safety and pharmacokinetic profiles as observed in adults. CAS + IMD was associated with low rates of COVID-19-related medically attended visits.

## BACKGROUND

For most children infected with severe acute respiratory syndrome coronavirus (SARS-CoV-2), the disease course is mild, however, for some, it can lead to severe illness, multisystem inflammatory syndrome, and even death. Age < 2 years and underlying comorbidities (eg, chronic lung disease, obesity, and immunocompromised conditions) are associated with an increased risk for severe coronavirus disease 2019 (COVID-19) [1, 2], highlighting the need for safe and effective therapeutics.

In adults, casirivimab and imdevimab (CAS + IMD), a combination of 2 non-competing recombinant monoclonal antibodies (mAbs) targeting the receptor- binding domain of the SARS-CoV-2 spike protein [3], was well-tolerated and reduced SARS-CoV-2 viral load, COVID-19 hospitalizations or all-cause death, and time to symptoms resolution versus placebo [4, 5]. However, the emergence of SARS-CoV-2 variants conferring a loss of activity for the previously approved or authorized anti-SARS-CoV-2 mAbs, including CAS + IMD, has limited the utility of these mAbs in COVID-19.

Next-generation anti-SARS-CoV-2 mAbs are in development to address ongoing unmet needs; therefore, it is important to share data informing mAb use in high-risk pediatric patients.

Herein, we present safety, pharmacokinetic (PK), and immunogenicity data from the pediatric cohort of a study evaluating CAS + IMD in high-risk outpatients with COVID-19, prior to the widespread circulation of Omicron-lineage and later variants.

## METHODS

### Trial Design

Study COV-2067 (NCT04425629) was an adaptive, multicenter (United States and Mexico), randomized, double-blind, placebo-controlled phase 1/2/3 trial evaluating the efficacy and safety of CAS + IMD in outpatients with ≥ 1 risk factor for severe COVID-19. The primary phase 3 analysis in adults was previously published [5]. Here, we describe the results from the pediatric cohort of the phase 3 portion. Pediatric participants were aged < 18 years with confirmed COVID-19 and ≥ 1 risk factor for severe COVID-19 (see Supplementary Methods and Supplementary Table 1). Participants were initially randomized 1:1:1 to receive a single intravenous dose of CAS + IMD 1200 mg, 2400 mg (or equivalent doses based on pediatric baseline bodyweight category; Supplementary Table 2), or placebo (Supplementary Figure 1). On February 24, 2021, the placebo arm was discontinued per recommendation from the Independent Data Monitoring Committee, and participants were subsequently randomized 1:1 to the CAS + IMD doses until a further change required all participants receive CAS + IMD 1200 mg.

Participants underwent randomization between January and December 2021. The Omicron variant (B.1.1.529) emerged in December 2021 [6], against which CAS + IMD showed reduced potency. The proportion of participants enrolled per month is shown in Supplementary Figure 2.

### Endpoints

The primary safety endpoints assessed the proportion of participants with treatment-emergent serious adverse events (SAEs) and hypersensitivity reactions (HSRs; grade ≥ 2) through day 29 and infusion-related reactions (IRRs; grade ≥ 2) through day 4. Grade 3 and 4 treatment-emergent adverse events (TEAEs) were collected through day 29, as were any TEAEs that led to a medically attended visit (MAV), to contextualize clinical events. All participants were followed to day 169 (end of study) for longer-term safety assessment. Since the collection of all TEAEs from participants would have imposed a significant burden on an already overstrained healthcare system, only targeted categories of TEAEs were collected to provide the most relevant safety information for evaluating the safety and tolerability of CAS + IMD. TEAEs voluntarily reported outside the targeted categories were also included.

PK, virologic, and clinical endpoints, as well as statistical analyses, are described in Supplementary Methods.

## RESULTS

### Baseline Characteristics

Of 206 pediatric participants assigned to treatment (Supplementary Figure 3), 192 (93%) had detectable SARS-CoV-2 and were included in the efficacy analysis set (modifed full analysis set [mFAS]). Nearly all (*n* = 191; 99.5%) received CAS + IMD; only 1 received placebo. Most participants (*n* = 121) received CAS + IMD 1200 mg IV because the 2400 mg arm (*n* = 70) was discontinued, leading to minor differences in cohort demographics by treatment group. In the mFAS, the median age was 11 years (51% were aged < 12 years); 56% were male; 63% were Hispanic or Latino; and 87% were White (Supplementary Table 3). The most common risk factors were chronic lung disease, including asthma (54.7%), and obesity (36.5%) (Supplementary Table 4).

Most participants (78.1%) were SARS-CoV-2 seronegative at baseline. The median (Q1:Q3) time from symptom onset to randomization was 3.0 (2.0:4.0) days, and the mean baseline viral load (standard deviation) was 6.85 (1.73) log_10_ copies/mL, with higher mean viral loads observed in those who were seronegative compared with those who were seropositive (Supplementary Table 3).

### Safety

Safety is presented for 202 pediatric participants who received the study drug (Table 1 and Supplementary Table 4).

**Table 1.** TEAEs to Day 169 (Pediatric Participants, SAF)

*N* (%)	Placebo (*n* = 2)	CAS + IMD 1200 mg IV (*n* = 129)	CAS + IMD 2400 mg IV (*n* = 71)	CAS + IMD combined (*N* = 200)
Total number of TEAEs^a^	1	84	37	121
Total number of grade 3 or 4 TEAEs	0	12	0	12
Total number of TE SAEs	0	4	0	4
Total number of TE AESIs^b^	0	12	5	17
Participants with any TEAE	1 (50.0)	23 (17.8)	12 (16.9)	35 (17.5)
Participants with any grade 3 or 4 TEAE	0	3 (2.3)	0	3 (1.5)
Participants with any TE SAE	0	3 (2.3)	0	3 (1.5)
Participants with any TE AESI	0	10 (7.8)	5 (7.0)	15 (7.5)
Participants with at least one TE AESI of infusion-related reaction (grade ≥ 2), through day 4^c^	0	0	1 (1.4)	1 (0.5)
Participants with at least one TE AESI of hypersensitivity reaction (grade ≥ 2), through day 4	0	1 (0.8)	0	1 (0.5)
Participants with TE AESIs that led to a MAV, through day 29	0	9 (7.0)	4 (5.6)	13 (6.5)
COVID-related	0	2 (1.6)	1 (1.4)	3 (1.5)
Non-COVID-related	0	7 (5.4)	3 (4.2)	10 (5.0)
Participants with any TEAE leading to death	0	0	0	0
Participants with any TEAE leading to withdrawal from the study	0	0	0	0
Participants with any TEAE leading to study infusion interruption^d^	0	0	0	0
Participants with any TEAE leading to study infusion discontinuation^e^	0	1 (0.8)	0	1 (0.5)

Notes: The SAF comprised all randomized participants who received any study drug (active or placebo).

Abbreviations: AESI, adverse event of special interest; CAS + IMD, casirivimab and imdevimab; IV, intravenous; MAV, medically attended visit; SAE, serious adverse event; SAF, safety analysis set; TE, treatment-emergent; TEAE, treatment-emergent adverse event.

^a^TEAEs collected include TE SAEs, AESIs, and grade 3 or 4 TEAEs as well as ad-hoc/voluntarily reported TEAEs by some sites.

^b^AESIs include grade ≥ 2 infusion-related reactions up to day 4, grade ≥ 2 hypersensitivity reactions up to day 29, and, to contextualize clinical events, any TEAE that led to an MAV up to day 29.

^c^TEAEs deemed related to treatment as per investigator assessment.

^d^Infusion interruption: the administration of the infusion was interrupted before being completed, but was subsequently re-started and the full planned dose was administered.

^e^Infusion discontinuation: the administration of the infusion was stopped before being completed, and the full planned dose was not administered.

The proportion of pediatric participants with targeted TEAEs or adverse events of special interest (AESIs) was generally balanced across the treatment groups, with minor discrepancies likely driven by differences in sample size. There were no TEAEs leading to death or study withdrawal.

There were 4 SAEs in total across 3 participants (1.5%) in the 1200 mg group (Table 1). An evaluation of SAEs reported by time to onset, to day 29, and from day 30 until the last available timepoint, did not show any specific safety trends: 2 occurred up to day 29 (metapneumovirus pneumonia and urinary retention) and 2 occurred after day 29 (suicidal ideation and respiratory distress). None were considered related to the study drug; all were confounded by the participant’s medical history. Details of grade 3 and 4 TEAEs are included in Supplementary Results. None were considered related to the study drug.

AESIs (grade ≥ 2 IRRs to day 4 or grade ≥ 2 HSRs to day 29) were experienced in 2 participants: 1 participant in the 2400 mg group experienced a grade 2 IRR of pyrexia, and 1 participant in the 1200 mg group experienced a grade 2 HSR of urticaria that resulted in discontinuation of study drug (Supplementary Table 4). Other AESIs, which included any TEAE that led to an MAV through day 29, were experienced by 13 (6.5%) participants (Supplementary Table 4). Further details are included in Supplementary Results.

Overall, both doses of CAS + IMD were well tolerated in pediatric participants, and the safety profile was consistent with results reported for adults.

### Virologic and Clinical Outcomes

While most (> 99%) participants received CAS + IMD, limiting the ability to make any meaningful conclusions regarding treatment effects, CAS + IMD 1200 mg and 2400 mg were associated with similar viral load reductions, consistent with the absence of any dose-responsive effect (Supplementary Figure 4).

Key clinical assessments showed no deaths, no COVID-19–related hospitalizations, and a low rate (1.5%; 3/202) of COVID-19–related MAVs (see Supplementary Results).

### Clinical Pharmacokinetics and Immunogenicity

**Figure 1.**
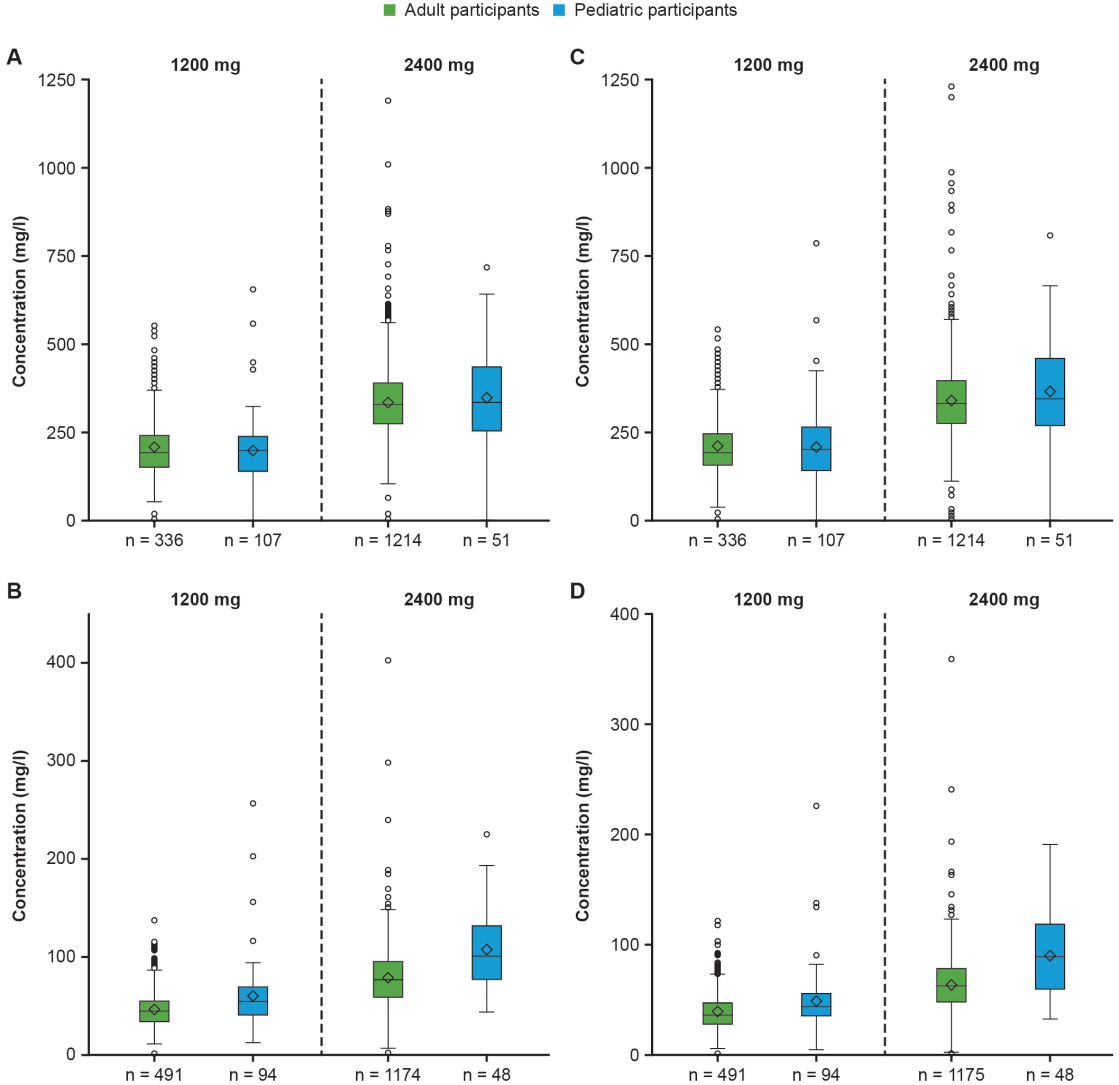
Concentrations of total CAS in serum at (A) the end of the infusion and (B) day 28, and total IMD in serum at (C) the end of the infusion and (D) day 28 by dose (pediatric participants, PKAS)^a^. Abbreviations: CAS, casirivimab; IMD, imdevimab; IV, intravenous; PKAS, pharmacokinetics analysis set. ^a^Pediatric participants received a single IV dose of CAS + IMD 1200 mg, 2400 mg, or equivalent based on pediatric baseline body-weight category; see also Supplementary Table 2.

CAS, IMD, and CAS + IMD (total drug) concentrations in serum increased dose-proportionally (Supplementary Figure 5) from 1200 mg to 2400 mg equivalent doses in pediatric participants (Supplementary Table 2). Total concentrations of each mAb at the end of infusion as well as on day 28 were similar between adult participants receiving 1200 mg and pediatric participants receiving a bodyweight equivalent dose (Figure 1).

There were no associations observed between change in viral load (time-weighted average change from baseline or change from baseline in viral load) and CAS + IMD combined concentration in serum on day 28 across different baseline viral load categories in this pediatric cohort (Supplementary Figure 6).

In the active treatment groups, 1 participant (0.5%) had treatment-emergent anti-drug antibodies (ADAs) against both CAS and IMD; both were low maximal titer (< 100). This participant was negative for neutralizing antibodies (NAbs) against CAS but positive for NAbs against IMD. No meaningful differences in the concentrations of CAS or IMD in serum were observed in this participant.

## DISCUSSION

Randomized, controlled data informing the use of anti-SARS-CoV-2 mAbs in pediatric outpatients are needed; here, we present the largest prospective study to date of pediatric outpatients treated with anti-SARS-CoV-2 mAbs.

In pediatric outpatients with COVID-19 and ≥ 1 risk factor for severe disease, treatment with CAS + IMD was well-tolerated, with a low incidence of grade ≥ 2 IRRs and HSRs, consistent with observations in high-risk adult outpatients [5]. TEAEs that led to an MAV were considered not related to the study drug, and most were mild-to-moderate and not COVID-19–related. Notably, the lack of placebo limits comparison to the number and type of adverse events that may have occurred in untreated participants.

Bodyweight tier-based dosing of CAS + IMD in pediatric outpatients resulted in similar CAS + IMD concentrations in serum and a similar concentration-nasopharyngeal viral load response relationship to that observed in adults. The rate of treatment-emergent ADAs against CAS and IMD was low, with no meaningful differences in serum drug concentration when immunogenicity was observed.

Conclusions with regard to the treatment effect in the pediatric cohort were limited as the placebo arm was discontinued soon after enrollment of pediatric participants commenced. Importantly, there were no deaths, no COVID-19–related hospitalizations, and low rates of COVID-19–related MAVs.

This study had some limitations. The sample size was relatively small and included only 1 participant in the placebo arm, limiting any meaningful conclusions regarding the treatment effect. The exclusion of participants vaccinated against COVID-19, and most participants being seronegative at baseline limits generalizability to the current situation where >95% of the world's population are seropositive [7].

Despite these limitations, the findings provide meaningful data to inform the dosing and development of next-generation mAb-based therapeutics for pediatric outpatients with COVID-19. This study affirms that body weight-tiered dosing of CAS + IMD was generally well-tolerated in high-risk pediatric participants, potentially supporting earlier-stage exploration of SARS-CoV-2 mAbs in pediatric outpatients with residual unmet need.

## Supplementary Data

Supplementary materials are available at the *Journal of The Pediatric Infectious Diseases Society* online (http://jpids.oxfordjournals.org).

piae105_suppl_Supplementary_Tables_1-4_Figures_1-6

## Data Availability

Qualified researchers may request access to study documents (including the clinical study report, study protocol with any amendments, blank case report form, and statistical analysis plan) that support the methods and findings reported in this manuscript. Individual anonymized participant data will be considered for sharing once the indication has been approved by a regulatory body if there is legal authority to share the data and there is not a reasonable likelihood of participant re-identification. Submit requests to https://vivli.org/.

## Notes

***Financial support.*** Supported by Regeneron Pharmaceuticals, Inc., and F. Hoffmann-La Roche Ltd. Certain aspects of this project have been supported in whole or in part with federal funds from the Department of Health and Human Services; Administration for Strategic Preparedness and Response; Biomedical Advanced Research and Development Authority, under OT number: HHSO100201700020C. The findings and conclusions herein are those of the authors and do not necessarily represent the views of the Department of Health and Human Services or its components.

***Potential conflicts of interest.*** T.D.N., M.T., S.G., S.A., J.C., A.W., J.X., J.D.D., S.C.I., C.P., D.A., M.H., D.S., G.P.G., E.C., N.B., P.D., B.K., R.B., A.T.D., and D.H. are employees and stockholders at Regeneron Pharmaceuticals, Inc. K.C.T., J.D.H., and G.A.H. are employees and stockholders of Regeneron Pharmaceuticals, Inc., and have a patent pending, which has been licensed and is receiving royalties, with Regeneron Pharmaceuticals, Inc. A.T.H. is an employee/stockholder of Regeneron Pharmaceuticals, Inc.; a stockholder of Pfizer; and reports a patent pending, which has been licensed and receiving royalties, with Regeneron Pharmaceuticals, Inc. L.R.-G. reports no conflicts of interest. A.K. reports support to their institution from Regeneron Pharmaceuticals, Inc., Vir, Gilead, Astra-Zeneca and Eli-Lilly; and advisory board participation for Gilead. E.M. reports payments to his institution received from SciClone Pharmaceuticals, Regeneron Pharmaceuticals, Inc., Pfizer, Chemic Labs/KODA Therapeutics, Cidara, and Leidos Biomedical Research Inc./NCI; and advisory board participation for Basilea; and grants from NIH/NIAID, NIH/NIGMS, and BARDA.
