# Supplementary material for: Safety and Pharmacokinetics of Casirivimab and Imdevimab (CAS + IMD) in Pediatric Outpatients With COVID-19
Source: J Pediatric Infect Dis Soc. 2024 Oct 1;13(11):589–93. doi: 10.1093/jpids/piae105 (PMC11599144; doi:10.1093/jpids/piae105)
Supplement: piae105_suppl_Supplementary_Tables_1-4_Figures_1-6 [file piae105_suppl_supplementary_tables_1-4_figures_1-6.docx]

Supplementary Appendix

# Study Sites and Investigators

**Advanced Pulmonary Research Institute, Loxahatchee, FL:** Neal Warshoff, Liudmila Moreiras

**AGA Clinical Trials, Miami, FL:** Dario Altamirano, Dickson Ellington, Faisal Fakih

**Arizona Liver Health, Tucson, AZ:** Anita Kohli, Vicki McIntyre, Yessica Sachdeva, Ashley Carney

**Arizona Liver Health, Puyallup, WA:** Yessica Sachdeva, Anita Kohli, Amanda McFarland, Dina Gibson, Victorine Ekoko

**Ark Clinical Research, Long Beach, CA:** Kenneth Kim, Lisa Neinchel, Nayna Paryani, Amber Mottola, Eva Day, Martha Navarro, Apinya Vutikullird

**Atella Clinical Research La Palma, CA:** Rafaelito Victoria, Xanthe Victoria, Rene Uong

**Atrium Health, Charlotte, NC:** Christopher Polk, Mindy Sampson, Michael Leonard, Lewis McCurdy, Leigh Ann Medaris, Zainab Shahid, Lisa Davidson

**Avera McKennan Hospital and University Health Center, Sioux Falls, SD:** Jawad Nazir, John Lee, Amy Elliott, Touba Naim, Khizar Hamid, Muhammad Hamza, Robert Kessler, Kara Bruning

**Axces Research, Sante Fe, NM:** Linda Gorgos, Erika Benson, Michael Palestine

**Bio-Medical Research, LLC, Miami, FL:** Lilia Roque-Guerrero, Ana Gomez Ramirez, Javier Capote, Gisel Paz

**Carolina Medical Research, Clinton, SC:** Nancy Patel, Ravikumar Patel, Ryan Sattar

**Catalina Research Institute, Montclair, CA:** Rizwana Mohensi, Shelia De Jesus-Maranan, Cecilia Casaclang

**Centex Studies, Lake Charles, LA:** Michael Seep, Celeste Brown, Joshua Whatley

**Chicago Clinical Research Institute, Chicago, IL:** Dennis Levinson, Norman James, Saad Alvi, Azazuddin Ahmed

**Clinical Research of Central Florida, Winter Haven, FL:** Robinson Koilpillai, Stephanie Cassady, Jennifer Cox, Eduardo Torres

**Crossroads Clinical Research, Corpus Christi, TX:** Michael Winnie, Jerry Plemons, Omesh Verma, Richard Leggett

**DM Clinical Research/BFHC Research, San Antonio, TX:** Ramon Reyes, Keith Beck, Brian Poliquin

**DM Clinical Research/LinQ Research, LLC, Pearland, TX:** Murtaza Mussaji, Jignesh Shah

**Duke Clinical Research, Durham, NC:** John Eppensteiner, Alexander Limkakeng, Joseph Borawski, Samuel Francis, Charles Gerardo, Emily Thatcher, Harajeshwar Kohli, Rachel O’Brian

**EME RED Hospitalaria, Mérida, Mexico:** Rodrigo Buendia, Natalia Romero Pavía, Juan Francisco Rubio Suárez, Mario Humberto Bustillos Pech

**Epic Medical Research, Red Oak, TX:** Haresh Boghara, Sunny Patel, Bari Eichelbaum

**Eukarya Pharmasite S.C., Nuevo Leon, Mexico:** Ricardo Tellez, Stephani Moreno

**Excel Clinical Research, Las Vegas, NV:** Duane Anderson, Sean Su, Alexander Akhavan, Diana Kirby, Joy Venglik, Crista Fedora

**FAICIC S. de RL de C.V., Veracruz, Mexico:** Alejandro Quintín Barrat Hernández, Silvano Omar Martinez Pérez, Edgar Iván Muñoz López

**Florida Pulmonary Research Institute, LLC, Winter Park, FL:** Faisal A. Fakih, Faisal M. Fakih, Fernando Alvarado, Daniel Layish, Jose Diaz, Andres Perez

**Fred Hutchinson Cancer Research Centre, Seattle, WA:** Michelle Karuna, Michael Boeckh, Elizabeth Church, Alison Roxby,

**Future Innovative Treatments, LLC, Colorado Springs, CO:** Bhaktasharan Patel, Gary Tarshis, Katrina Grablin

**Global Clinical Professionals Research, St Petersburg, FL:** Roxana Stoici, Gualberto Perez, Joseph Pica, Enrique Villareal

**Harlem Hospital Center – NYCHHC, Harlem, NY:** Farbod Raiszadeh, Sharon Mannheimer, Khaing Myint, Hussein Assallum, Lovelyamma Varghese, Akari Kyawa

**Holy Name Medical Center, Teaneck, NJ:** Suraj Saggar, Thomas Birch, Benjamin De La Rosa, Karyna Neyra, Erina Kunwar

**Hope Clinical Research, Canoga Park, CA:** Hessam Aazami, Cheryl Bland, Deborah Wu, Jamsheed Akhavan

**Hospital Angeles Chihuahua, Chihuahua, Mexico:** Belinda Sofia Gomez Quintana, Hector Rascón Marquez

**Houston Methodist Hospital, Houston, TX:** Howard Huang, Jihad Georges Youssef, Simon Yau, Ahmad Goodarzi, Mukhtar Al-Saadi, Faisal Zahiruddin

**IACT Health, Columbus, GA:** Jeffrey Kingsley, April Pixler

**Icahn School of Medicine at Mount Sinai, Manhattan, NY:** Judith Aberg, Michelle Cespedes, Alexandra Abrams-Downey, Erna Kojic, Luz Lugo, Sean Liu, Nadim Salomon, David Perlman, Deena Altman, Farah Rahman, Georgina Osorio, Joseph Mathew, Sanjana Koshy, Dana Mazo, Francesca Cossarini, Sondra Middleton, Alina Jen, Erika Maria Reategui Schwarz

**Innova Health Care Services (INOVA Fairfax Hospital), Falls Church, VA:** Christopher deFilippi, Steven Nathan, Lindsay Clevenger

**Instituto de Investigaciones Clínicas para la Salud A.C. Durango, Mexico:** Isabel Buendia Suarez, Cristina Resendez

**Jacobi Medical Center, Bronx, NY:** Gabriele DeVos, David Stein, Jason Leider, Kellie Roe, Jane Devereux, Elizabeth Jenny-Avital

**Köhler and Milstein Research SA de CV, Merida, Yucatan, Mexico:** Jesus Abraham Simon Campos, Felipe de Jesús Pineda Cárdenas

**Lincoln Medical Center – NYCHHC, Bronx, NY:** Vidya Menon, Moiz Kasubhai, Usha Venugopal, Anjana Pillai, Franscene Oulds, Paola Carugno, Daniel Sittler

**Long Beach Medical Center, Long Beach, CA:** Jimmy Johannes, Thomas Jaing, Christopher Yee, Henry Su, Andrew Wittenberg, Anthony Arguija

**Maryland School of Medicine, Baltimore, MD:** Richard Wilkerson, Shyam Kottilil, Shivakumar Narayanan, Joel Chua, Jennifer Husson, John Baddley

**Medical Research of Westchester, Miami, FL:** Richard Perez-Perez, Carlos J. Bello, Esperanza Arce-Nunez, Jorge Acosta, Julio L. Arronte

**Medical University of South Carolina, Charleston, SC:** Eric Meissner, Patrick Flume, Andrew Goodwin, Deeksha Jandhyala, Nandita Nadig

**Mercury Clinical Research, Houston, TX:** Rajasekaran Annamalai, Huy Nguyen, Nizar Nayani, Mahalakshmi Ramchandra

**META Medical Research Institute, Dayton, OH:** Priyesh Mehta, Jacqueline Horne, Grace Hassan

**Miami Valley Hospital, Dayton, OH:** Thomas Herchline, Steve Burdette, Jonathan Pope, David Herman

**Midland Florida Clinical Research Center, Deland, FL:** Godson Oguchi, DeAndrea Duffus

**Midway Immunology and Research Center, Fort Pierce, FL:** Moti Ramgopal, Brenda Jacobs, Lisa Cason, Angela Trodglen

**Next Level Urgent Care, Houston, TX:** Terence Chang, Robbyn Traylor, Lenee Gordon, John McDivitt, Lizette Castro

**Palos Verdes Medical Group (PVMG), Peninsula Research Associates, Inc., Rolling Hills Estates, CA:** Lawrence Sher, Monica Saad, LeighAnn Schmidt

**Pharma Tex Research, LLC, Amarillo, TX:** David Brabham, Mark Sigler, Tarek Naguib

**PMG Research of McFarland Clinic, Ames, IA:** Jennifer Killion, Rupal Amin, Shauna Basener, Timothy Lowry

**PMG Research of Wilmington, Wilmington, NC:** Kevin Cannon, Mesha Chadwick

**Providence Saint John’s Health Center, Santa Monica, CA:** Terese Hammond, Fabian Andres Romero, Steven O’Day, Trevan Fischer, Ana Rocha, Anmol Rangoola

**Qway Research, Hialeah, FL:** Oscar Galvez, Fausto Castillo

**Regional One Health, Puyallup, WA and Memphis, TN:** John Jefferies, Scott Strome, Sandy Arnold, Terri Finkel, Amber Thacker, Amik Sodhi, Elisha McCoy, Daniel Wells, Nathaniel Rogers, David Schwartz

**Remington-Davis, Columbus, OH:** Edward Cordasco, Brian Zeno, Heather Holmes, Heather Lee

**Rhode Island Hospital, Providence, RI:** Eleftherios Mylonakis, Dimitrios Farmakiotis, Natasha Ryback, Karen Tashima, Francesca Beaudoin, Selim Suner, Gregory Jay, Katelyn Moretti, Adam Aluisio, Naz Karim, Sonya Naganathan, William Binder, Adam Levine, Neel Belani, John Lee, Taneisha Wilson, Anshul Parulkar, Ramu Kharel, Alexis Lawrence

**RM Pharma Specialists S.A. de Colonia del Valle, Mexico City, Mexico:** Lucero Sanchez, Ana Karla Guzmán Romero

**Ruane Clinical Research Group, Los Angeles, CA:** Peter Ruane, Peter Wolfe, Kenny Trinidad, Isaac Berlin

**San Francisco Research Institute, San Francisco, CA:** Mark Savant, Francis Hsiao, Edna Yee

**Sarasota Memorial Hospital, Sarasota, FL:** Manuel Gordillo, Rishi Bhattacharyya, Sudha Tallapragada, Annette Artau, Julie Larkin, Roberto Mercado, Michael Milam, Natan Kraitman, Sarah Temple, Lenka Offner, Rabih Loutfi, Kirk Voelker, Michael Lowry, Marshall Frank, Ashley Grant

**SignatureCare Emergency Center – TC Jester, Houston, TX:** Alan Skolnick, Harold Minkowitz, David Leiman, Todd Price, Anatoli Krasko

**Stanford University, Palo Alto, CA:** Upinder Singh, Aruna Subramanian, Yvonne Maldonado, Jason Andrews, Chaitan Khosla

**Sun Research Institute, San Antonio, TX:** Carl Dukes, Robert Bass, Larry Lothringer, Leonel Reyes

**Tandem Clinical Research, Maitland, FL:** Esteban Olivera, Mayra Abreu

**Tandem Clinical Research, Marrero, LA:** Adil Fatakia, Marissa Miller, Kristen Clinton, Gary Reiss

**Temple University Hospital (TUH), Philadelphia, PA:** Gerard Criner, Nathaniel Marchetti, Parag Desai, Daniel Salerno, Fredric Jaffe, Samuel Krachman, Matthew Zheng, Maulin Patel, Junad Chowdhury, Daniel Mueller

**The George Washington University Hospital, Washington, WA:** David Diemert, Afsoon Roberts, David Parenti, Hana Akselrod, Marc Siegel, Andrew Meltzer, Elissa Malkin, Gary Simon

**Triple O Research Institute PA, West Palm Beach, FL:** Olayemi Osiyemi, Jose A. Menajovsky-Chaves, Christina Campbell

**Tulane University School of Medicine, New Orleans, LA:** Dahlene Fusco, Arnaud Drouin, Joshua Denson, Jerry Zifodya, Christine Bojanowski, Monika Dietrich, Stacy Drury

**Universal Medical and Research Center, LLC, Miami, FL:** Gerard Acloque**,** Agustin Martinez

**University of California (UC) Davis, Sacramento, CA:** Timothy Albertson, Nicholas Kenyon, Brian Morrissey, Christian Sandrock, Stuart Cohen

**University of Iowa, Iowa City, IA:** Alejandro Comellas, Joel Kline, Spyridon Fortis

**University of Mississippi Medical Center, Jackson, MS:** Gailen Marshall, Utsav Nandi, Vishnu Garla, John Spurzem, Andrew Wilhelm

**University of South Florida, Tampa, FL:** Kami Kim, Seetha Lakshmi, Tiffany Vasey, Asa Oxner, Jason Wilson, Lucy Guerra

**University of Texas (UT) – Southwestern Medical Center, Dallas, TX:** Satish Mocherla, Mamta Jain, Jessica Meisner, Nancy Rollins

**Wellstar Kennestone Hospital, Marietta, GA:** Danny Branstetter, Neha Paranjape

**Willis-Knighton Physician Network, Shreveport, LA:** Joseph Bocchini, Clint Wilson

**Xera Med Research, Boca Raton, FL:** Anna Martin, Gargi Gharat, Candace Kokaram, Ket Wray, Clement Partap, Ulyana Arzamasova, Kristina Louissaint, Maria Fernandez

**Xera Med Research, Miami, FL:** Anna Martin, Ket Wray, Kristina Louissaint, Maria Fernandez, Gargi Gharat

# Regeneron Study Team

Achint Chani, Adebiyi Adepoju, Adnan Mahmood, Aisha Mortagy, Ajla Dupljak, Alexander Kansky, Alison Brown, Alpana Waldron, Amanda Cook, Amy Froment, Andrea Hooper, Andrea Margiotta, Andrew Bombardier, Anne Smith, Aswani Bathula, Bari Kowal, Barry Siliverstein, Benjamin Horel, Bret Musser, Brian Bush, Brian Head, Bryan Zhu, Camille Debray, Careta Phillips, Carol Lee, Caryn Trbovic, Catherine Elliott, Chad Fish, Charlie Ni, Charlotte Lyon, Christina Perry, Christine Enciso, Christopher Caira, Christopher Chamak, Christopher Powell, Cliff Baum, Colby Burk, Crystal LaPoint, Cynthia Pan, Danise Subramaniam, David Liu, David Stein, Daya Gulabani, Deborah Leonard, Denise Bonhomme, Denise Kennedy, Derrick Bramble, Dhanalakshmi Barron, Diana Rofail, Dipinder Kaur, Dominique Atmodjo Watkins, Dona Bianco, Donna Gambaccini, Eduardo Forleo Neto, Edward Jean-Baptiste, Ehsan Bukhari, Elizabeth Bucknam, Emily Nanna, Esther Huffman O'Keefe, Evelyn Gasparino, Georgia Bellingham, Giane Sumner, Grainne Moggan, Grainne Power, Haitao Gao, Haixia Zeng, Hannah Smith, Heath Gonzalez, Helen Kang, Hibo Noor, Ian Minns, James Donohue, Janice Austin, Janie Parrino, Jeannie Yo, Jenna McDonnell, Jennifer Hamilton, Jessica Boarder, Jing Xiao, Jingchun Yu, Joanne Malia, Joanne Tucciarone, John Strein, Jonathan Cohen, Jordan Ursino, Joseph Im, Joseph Wolken, Karen Browning, Karen Yau, Kenneth Turner, Kimberly Dornheim, Kit Chiu, Kristina McGuire, Kristy Macci, Kurt Ringleben, Kyle Foster, Lacey Douthat, Laura Sarkis, Linda Kelly, Latora Knighton, Lisa Boersma, Lisa Hersh, Lisa Purcell, Lisa Sherpinsky, Lori Geissler, Mabel Osa-Joachimo, Martha Simpkins, Nagaratna Reddy Medapti, Nagendher Burra, Naresh Lall, Neena Sarkar, Nicholas Moore, Nicole Memblatt, Nikki Miocevic, Nirav Shah, Nitin Kumar, Nkechi Moghalu, Pallavi Rajput, Patricia Humphries, Pradeep Thanigaimani, Purushottam Risal, Rafia Bhore, Sara Dale, Sonia Yanes, Steven Chen, Suzanne Luther, Yuming Zhao

# Supplementary Methods

## Risk Factors

Risk factors included:

- Obesity (body mass index ≥95th percentile for age and sex based on Centers for Disease Control and Prevention growth charts in patients ≥2 years only)
- Cardiovascular disease (including hypertension)
- Chronic lung disease (including asthma)
- Type 1 or 2 diabetes mellitus
- Chronic kidney disease (including those on dialysis)
- Chronic liver disease
- Immunocompromised
- Any underlying genetic condition, neurologic condition, metabolic condition, or congenital heart disease deemed by the investigator to be a risk factor for severe COVID-19.

## Inclusion and Exclusion Criteria

Patients in this pediatric cohort were <18 years old, not pregnant at randomization, and non-hospitalized. All participants had confirmed COVID-19, with a positive SARS-CoV-2 test result ≤72 hours before randomization and symptom onset ≤7 days before randomization. Participants also had ≥1 risk factor for severe COVID-19

Participants were excluded if they had prior, current, or planned future use of COVID-19 convalescent plasma, monoclonal antibodies against SARS-CoV-2, intravenous immunoglobulin, systemic corticosteroids, or treatments for COVID-19 (investigational, authorized or approved), and prior, current, or planned (within 90 days of study drug administration or per current Centers for Disease Control and Prevention recommendations) of any authorized or approved vaccine for COVID-19.

## Pharmacokinetics

Concentrations of casirivimab (CAS) and imdevimab (IMD) and presence of anti-drug antibodies (ADAs) to CAS and IMD in serum were determined over time. Concentration-response for viral load reduction from day 1 to day 7, and similarity in concentration at comparable doses at the end of infusion and at day 29 between the pediatric and adult populations were also investigated. Serum samples were analyzed for total CAS and total IMD using a validated electrochemiluminescence immunoassay as previously described by Irvin et al [1]. The lower limit of quantitation was 0.156 mg/l in neat human serum. Immunogenicity was determined by measuring the presence of ADAs and neutralizing antibodies (NAbs) to CAS and to IMD. ADAs were determined in serum using validated electrochemiluminescence bridging immunoassays. The presence of NAbs was determined using validated competitive ligand-binding assays.

## Virologic and Clinical Endpoints

Secondary endpoints included the proportion of patients with ≥1 COVID-19-related MAV (by hospitalizations and emergency department [ED], urgent care, or physician’s office/telemedicine visits) or all-cause death through day 29. Virologic efficacy was assessed by evaluating the time-weighted average (TWA) change from baseline in viral load from day 1 to 7, and the change from baseline in viral load at each visit, measured by reverse transcription quantitative polymerase chain reaction (RT-qPCR) in nasopharyngeal (NP) samples. Time to COVID-19 symptoms resolution was evaluated as an exploratory endpoint for patients ≥12 years old using the 23-item Symptoms Evolution of COVID-19 (SE-C19) instrument [18], which assessed COVID-19 symptoms daily through day 29.

**Statistical Analysis**

The full analysis set (FAS) included all randomized participants; the modified FAS (mFAS) included all participants who had a positive SARS-CoV-2 RT-qPCR test in NP swab samples at randomization; the safety analysis set included all randomized participants who received any study drug; the PK analysis set (PKAS) included all participants who received active study drug and had ≥1 non-missing result following the first dose of the active study drug (PK samples for patients who received placebo treatment were not analyzed); the anti-drug antibody (ADA) analysis set included all participants who received any study drug and had ≥1 non-missing ADA result following their first dose, the ADA analysis set was based on actual treatment received, rather than as randomized; the concentration-response analysis set included the baseline RT-qPCR positive (mFAS) and seronegative PKAS and placebo-treated participants who were included in the seronegative mFAS that had ≥1 non-missing baseline, and one non-missing post dose viral load or clinical efficacy measurement. For the purposes of this concentration-response population, as samples from placebo patients were not assayed, all missing concentrations were imputed as 0.

Clinical efficacy endpoints were summarized descriptively. No multiplicity adjustment was applied.

# Supplementary Results

## Safety

Two participants (1200 mg group) experienced a total of three grade 3 or 4 TEAEs up to day 29. One was the aforementioned SAE of metapneumovirus pneumonia, which was grade 4; another participant experienced two transient grade 3 TEAEs of elevated alanine aminotransferase and aspartate aminotransferase on day 7. The liver enzyme elevations were confounded by underlying COVID-19 illness and recent ibuprofen use. CAS+IMD-drug-induced liver injury was not suspected as the liver function test abnormalities trended towards normalization within 1 week after detection while the study drug was expected to persist at therapeutic concentrations. None of the TEAEs were considered related to study drug.

Three participants (1.5%) experienced a COVID-19-related MAV through day 29 (one in the 1200 mg group had an ED visit for vomiting and pyrexia related to COVID-19 and two participants [one in the 1200 mg group and one in the 2400 mg group] had a physician office or telemedicine visit for worsening of COVID-19 congestion and/or cough). For the 10 who experienced non-COVID-19-related TEAEs that led to a MAV, events included conjunctivitis (n = 2), gastroenteritis, otitis externa, metapneumovirus pneumonia, injury, tonsillitis, Crohn’s disease flare, sinusitis (n = 2), and urinary retention. Each was considered unrelated to study drug, and all were considered mild or moderate (i.e. grade 1 or 2) except for the previously described grade 4 TEAE (metapneumovirus pneumonia).

Supplementary Table 1. Summary of risk factors for severe COVID-19 in pediatric patients (mFAS)

| **Risk factor for severe COVID-19** | **Placebo (n = 1)** | **CAS+IMD 1200 mg IV (n = 121)** | **CAS+IMD 2400 mg IV (n = 70)** | **CAS+IMD combined (n = 191)** | **Total (N = 192)** |  |
| --- | --- | --- | --- | --- | --- | --- |
| Obesity^a^ | 0 | 44 (36.4) | 26 (37.1) | 70 (36.6) | 70 (36.5) |  |
| Cardiovascular disease, including hypertension | 0 | 9 (7.4) | 2 (2.9) | 11 (5.8) | 11 (5.7) |  |
| Chronic lung disease, including asthma | 1 (100) | 69 (57.0) | 35 (50.0) | 104 (54.5) | 105 (54.7) |  |
| Type 1 or type 2 diabetes mellitus | 0 | 0 | 4 (5.7) | 4 (2.1) | 4 (2.1) |  |
| Chronic kidney disease, including those on dialysis | 0 | 0 | 0 | 0 | 0 |  |
| Chronic liver disease | 0 | 0 | 1 (1.4) | 1 (0.5) | 1 (0.5) |  |
| Immunocompromised^b^ | 0 | 7 (5.8) | 10 (14.3) | 17 (8.9) | 17 (8.9) |  |
| Immunosuppressed | 0 | 7 (5.8) | 10 (14.3) | 17 (8.9) | 17 (8.9) |  |
| Taking immunosuppressants | 0 | 0 | 0 | 0 | 0 |  |
| Any underlying genetic, neurologic, or metabolic condition, or congenital heart disease | 0 | 18 (14.9) | 8 (11.4) | 26 (13.6) | 26 (13.5) | |
| Age <12 months | 0 | 1 (0.8) | 0 | 1 (0.5) | 1 (0.5) | |

BMI, body mass index; CAS, casirivimab; IMD, imdevimab; IV, intravenous; mFAS, modified full analysis set.

^a^Obesity is defined as BMI (kg/m^2^) ≥95th percentile for age and sex based on Centers for Disease Control and Prevention growth charts for subjects <18 years old at randomization.

^b^Immunocompromised status was based on investigator assessment and may have been due to an underlying immunosuppressive condition (denoted as ‘immunosuppressed’) or the prolonged use of immune-weakening medications (denoted as ‘taking immunosuppressants’).

**Supplementary Table 2. CAS+IMD IV doses for each body weight group**

| **Body weight group** | **Dose equivalent for CAS+IMD 1200 mg IV dose (600 mg per mAb)** | **Dose equivalent for CAS+IMD 2400 mg IV dose (1200 mg per mAb)** |
| --- | --- | --- |
| ≥40 kg | 1200 mg (600 mg per mAb) | 2400 mg (1200 mg per mAb) |
| ≥20 kg to <40 kg | 450 mg (225 mg per mAb) | 900 mg (450 mg per mAb) |
| ≥10 kg to <20 kg | 224 mg (112 mg per mAb) | 450 mg (225 mg per mAb) |
| ≥5 kg to <10 kg | 120 mg (60 mg per mAb) | 224 mg (112 mg per mAb) |
| ≥2.5 kg to <5 kg | 60 mg (30 mg per mAb) | 120 mg (60 mg per mAb) |
| <2.5 kg | 30 mg (15 mg per mAb) | 60 mg (30 mg per mAb) |

CAS, casirivimab; IMD, imdevimab; IV, intravenous; mAb, monoclonal antibody.

Supplementary Table 3. Demographics and Baseline Characteristics (Pediatric Patients, mFAS)

|  | **Placebo (n = 1)** | **CAS+IMD 1200 mg IV (n = 121)** | **CAS+IMD 2400 mg IV (n = 70)** | **CAS+IMD combined (n = 191)** | **Total (N = 192)** |
| --- | --- | --- | --- | --- | --- |
| Age (years) |  |  |  |  |  |
| Mean (SD) | 16.0 (.) | 10.8 (4.12) | 12.4 (3.29) | 11.4 (3.91) | 11.4 (3.91) |
| Median | 16.0 | 11.0 | 12.0 | 11.0 | 11.0 |
| Q1 : Q3 | N/A | 8.0 : 14.0 | 10.0 : 16.0 | 9.0 : 14.0 | 9.0 : 14.0 |
| Age group, n (%) |  |  |  |  |  |
| <12 years | 0 | 68 (56.2) | 30 (42.9) | 98 (51.3) | 98 (51.0) |
| ≥12 to <18 years | 1 (100) | 53 (43.8) | 40 (57.1) | 93 (48.7) | 94 (49.0) |
| Sex, n (%) |  |  |  |  |  |
| Male | 0 | 57 (47.1) | 51 (72.9) | 108 (56.5) | 108 (56.3) |
| Female | 1 (100) | 64 (52.9) | 19 (27.1) | 83 (43.5) | 84 (43.8) |
| Ethnicity, n (%) |  |  |  |  |  |
| Hispanic or Latino | 0 | 82 (67.8) | 38 (54.3) | 120 (62.8) | 120 (62.5) |
| Not Hispanic or Latino | 1 (100) | 39 (32.2) | 30 (42.9) | 69 (36.1) | 70 (36.5) |
| Not reported | 0 | 0 | 2 (2.9) | 2 (1.0) | 2 (1.0) |
| Race, n (%) |  |  |  |  |  |
| White | 1 (100) | 108 (89.3) | 58 (82.9) | 166 (86.9) | 167 (87.0) |
| Black or African American | 0 | 7 (5.8) | 7 (10.0) | 14 (7.3) | 14 (7.3) |
| Asian | 0 | 1 (0.8) | 1 (1.4) | 2 (1.0) | 2 (1.0) |
| Unknown | 0 | 3 (2.5) | 2 (2.9) | 5 (2.6) | 5 (2.6) |
| Not Reported | 0 | 2 (1.7) | 2 (2.9) | 4 (2.1) | 4 (2.1) |
| Weight category, n (%) |  |  |  |  |  |
| <10 kg | 0 | 2 (1.7) | 0 | 2 (1.0) | 2 (1.0) |
| 10 to <20 kg | 0 | 12 (9.9) | 1 (1.4) | 13 (6.8) | 13 (6.8) |
| 20 to <40 kg | 0 | 29 (24.0) | 19 (27.1) | 48 (25.1) | 48 (25.0) |
| ≥40 kg | 1 (100) | 78 (64.5) | 50 (71.4) | 128 (67.0) | 129 (67.2) |
| Obesity, n (%)^a^ |  |  |  |  |  |
| No | 1 (100) | 77 (63.6) | 44 (62.9) | 121 (63.4) | 122 (63.5) |
| Yes | 0 | 44 (36.4) | 26 (37.1) | 70 (36.6) | 70 (36.5) |
| Time from symptom onset to randomization, days |  |  |  |  |  |
| Mean (SD) | 3.0 (.) | 2.6 (1.44) | 2.7 (1.42) | 2.7 (1.43) | 2.7 (1.43) |
| Baseline viral load, log_10_ copies/ml, mean (SD) | 4.50 (.) | 6.69 (1.67) | 7.17 (1.81) | 6.86 (1.73) | – |
| Baseline serology status, n (%)^b^ |  |  |  |  |  |
| Negative | 1 (100) | 93 (76.9) | 56 (80.0) | 149 (78.0) | 150 (78.1) |
| Positive | 0 | 21 (17.4) | 9 (12.9) | 30 (15.7) | 30 (15.6) |
| Other | 0 | 7 (5.8) | 5 (7.1) | 12 (6.3) | 12 (6.3) |

Abbreviations: BMI, body mass index; CAS+IMD, casirivimab and imdevimab; IV, intravenous; mFAS, modified full analysis set; N/A, not applicable; SD, standard deviation.

^a^Obesity is defined as BMI (kg/m^2^) ≥95th percentile for age and sex based on Centers for Disease Control and Prevention growth charts for subjects <18 years old at randomization.

^b^Serostatus was considered positive if any available anti-SARS-CoV-2 antibody test utilized was positive, negative if all available tests were negative, and other if serostatus was neither positive or negative (for example, borderline result) or was unknown.

Supplementary Table 4. Details of adverse events of special interest (SAF)

| **AESI** | **Preferred term** | **Event grade** | **Relationship to treatment** | **Treatment group** |
| --- | --- | --- | --- | --- |
| Grade ≥2 IRR to day 4 |  |  |  |  |
|  | Pyrexia | 2 | Related | CAS+IMD 2400 mg IV |
| Grade ≥2 HSR to day 29 |  |  |  |  |
|  | Urticaria | 2 | Related | CAS+IMD 1200 mg IV |
| TEAE with MAV not related to COVID-19 to day 29 |  |  |  |  |
|  | Conjunctivitis | 1 | Not related | CAS+IMD 1200 mg IV |
|  | Conjunctivitis | 2 | Not related | CAS+IMD 1200 mg IV |
|  | Crohn’s disease | 1 | Not related | CAS+IMD 2400 mg IV |
|  | Gastroenteritis | 2 | Not related | CAS+IMD 1200 mg IV |
|  | Injury | 2 | Not related | CAS+IMD 1200 mg IV |
|  | Metapneumovirus pneumonia | 4 | Not related | CAS+IMD 1200 mg IV |
|  | Otitis externa | 2 | Not related | CAS+IMD 1200 mg IV |
|  | Sinusitis | 1 | Not related | CAS+IMD 1200 mg IV |
|  | Sinusitis | 1 | Not related | CAS+IMD 2400 mg IV |
|  | Tonsilitis | 1 | Not related | CAS+IMD 2400 mg IV |
|  | Urinary retention | 1 | Not related | CAS+IMD 1200 mg IV |
| TEAE with MAV related to COVID-19 to day 29 |  |  |  |  |
|  | COVID-19 | 2 | Not related | CAS+IMD 1200 mg IV |
|  | COVID-19 | 2 | Not related | CAS+IMD 2400 mg IV |
|  | Pyrexia | 2 | Not related | CAS+IMD 1200 mg IV |
|  | Vomiting | 2 | Not related | CAS+IMD 1200 mg IV |

AESI, adverse event of special interest; CAS, casirivimab; HSR, hypersensitivity reaction; IMD, imdevimab; IRR, infusion-related reaction; IV, intravenous; MAV, medically attended visit; SAF, safety analysis set; TEAE, treatment-emergent adverse event.

Supplementary Figure 1. Study design


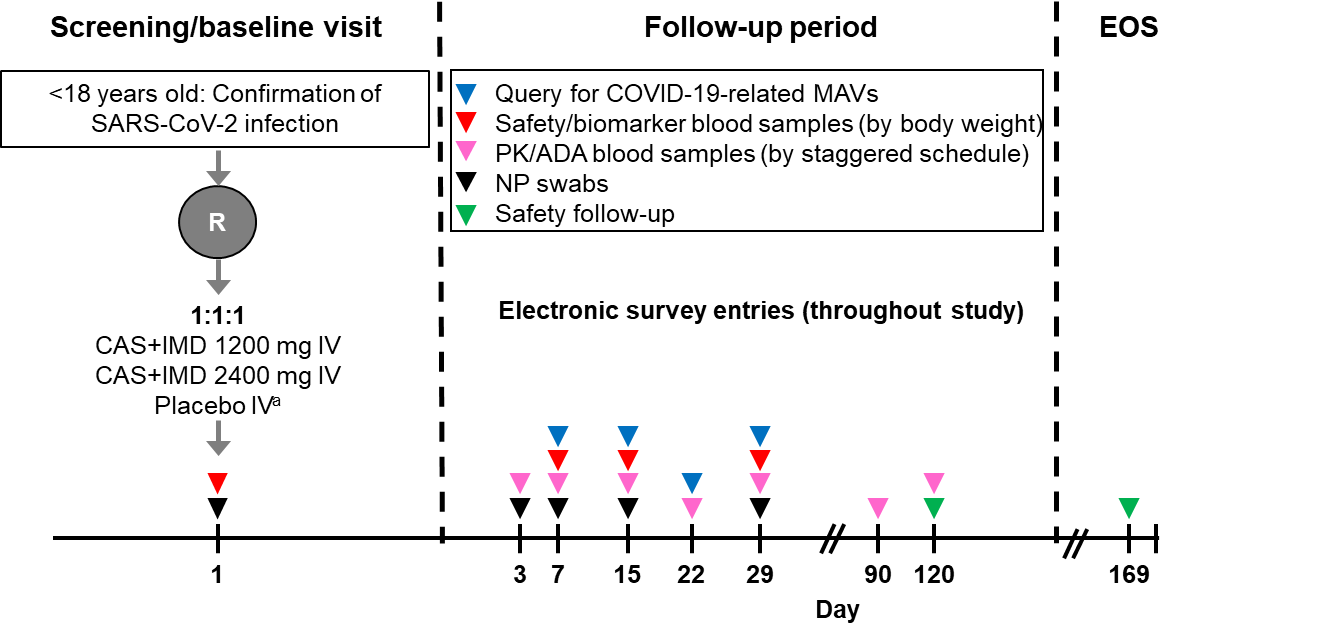


^a^No randomization to placebo as of 25 February 2021.

ADA, anti-drug antibody; COVID-19, coronavirus disease 2019; EOS, end of study; IV, intravenous; MAV, medically attended visit; NP, nasopharyngeal; PK, pharmacokinetic; R, randomization; SARS-CoV-2, severe acute respiratory syndrome coronavirus 2.

Supplementary Figure 2. Patient monthly enrollment during the study in 2021 (full analysis set)


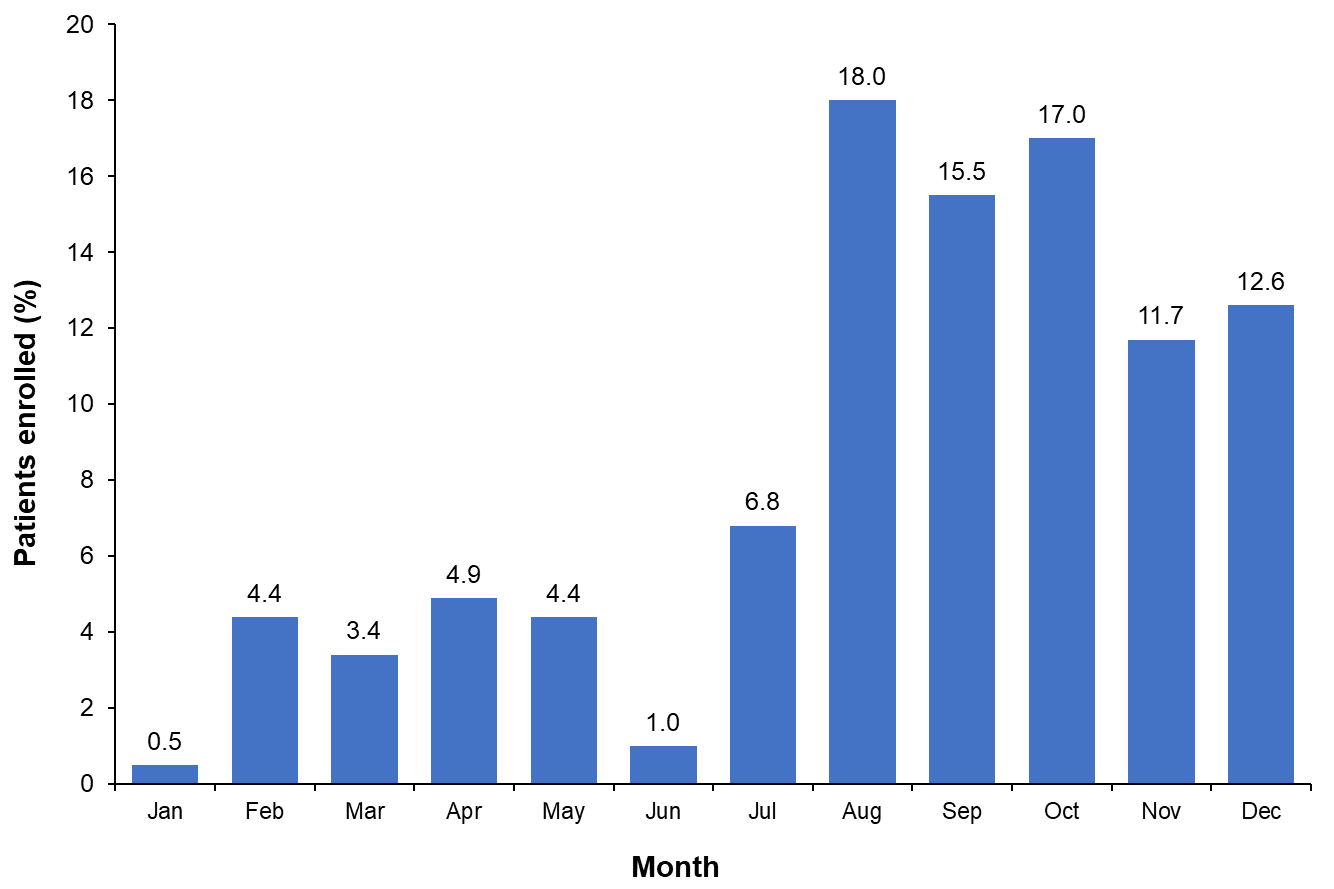


Supplementary Figure 3. CONSORT flow diagram (full analysis set)


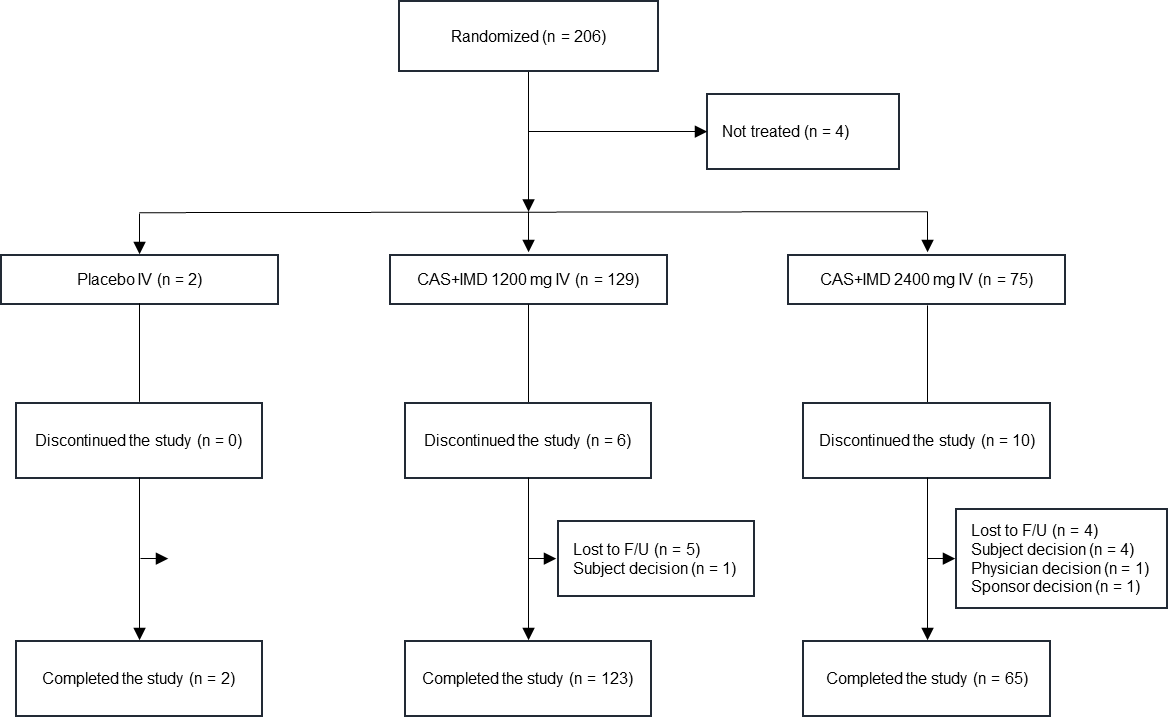


CAS+IMD, casirivimab and imdevimab; F/U, follow-up; IV, intravenous.

Patients in the full analysis set included all randomized patients and based on treatment allocated (as randomized). Patients in the modified full analysis set (mFAS; efficacy analysis) included all randomized patients with positive RT-qPCR in nasopharyngeal swab samples at randomization and based on treatment allocated (as randomized).

Supplementary Figure 4. Mean (±SE) viral load (log_10_ copies/ml) from day 1 at each visit up to day 29 in NP samples (pediatric patients, mFAS).


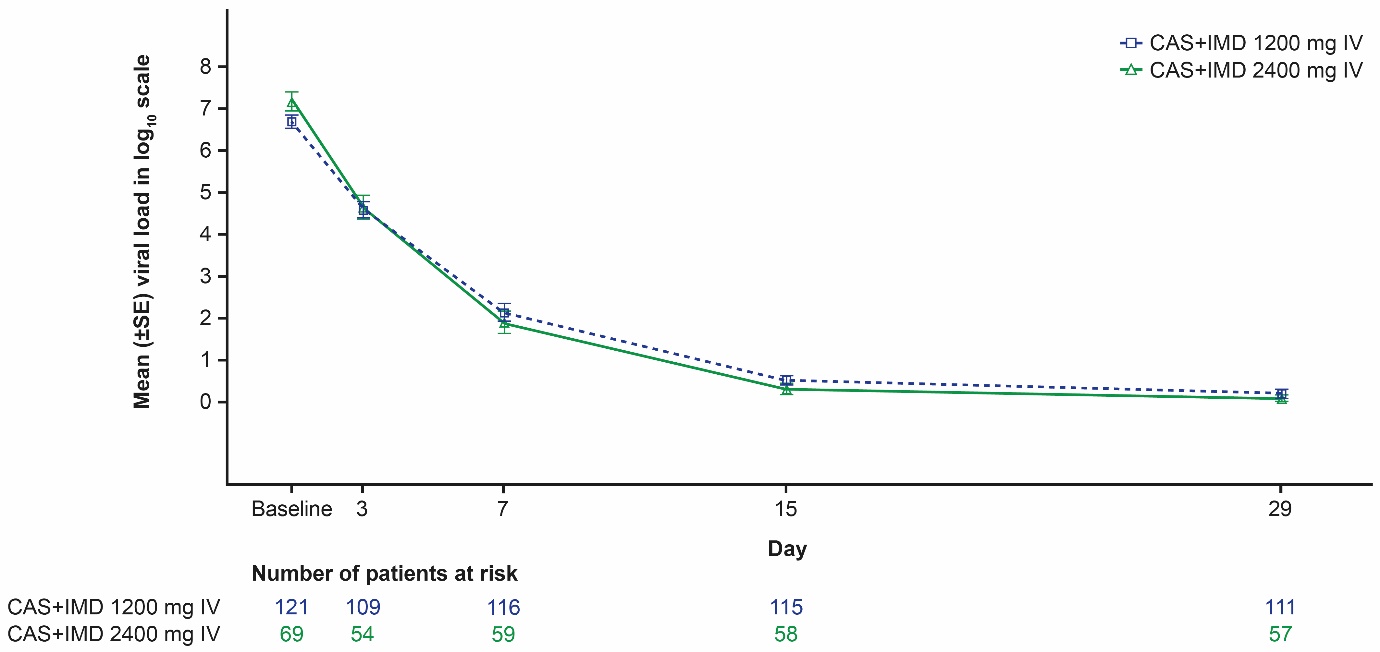


CAS+IMD, casirivimab and imdevimab; IV, intravenous; mFAS, modified full analysis set; NP, nasopharyngeal; SE, standard error.

Supplementary Figure 5. Mean (+SD) concentrations of total casirivimab, total imdevimab, and total CAS+IMD in serum by nominal time and treatment group in pediatric patients (pharmacokinetics analysis set)


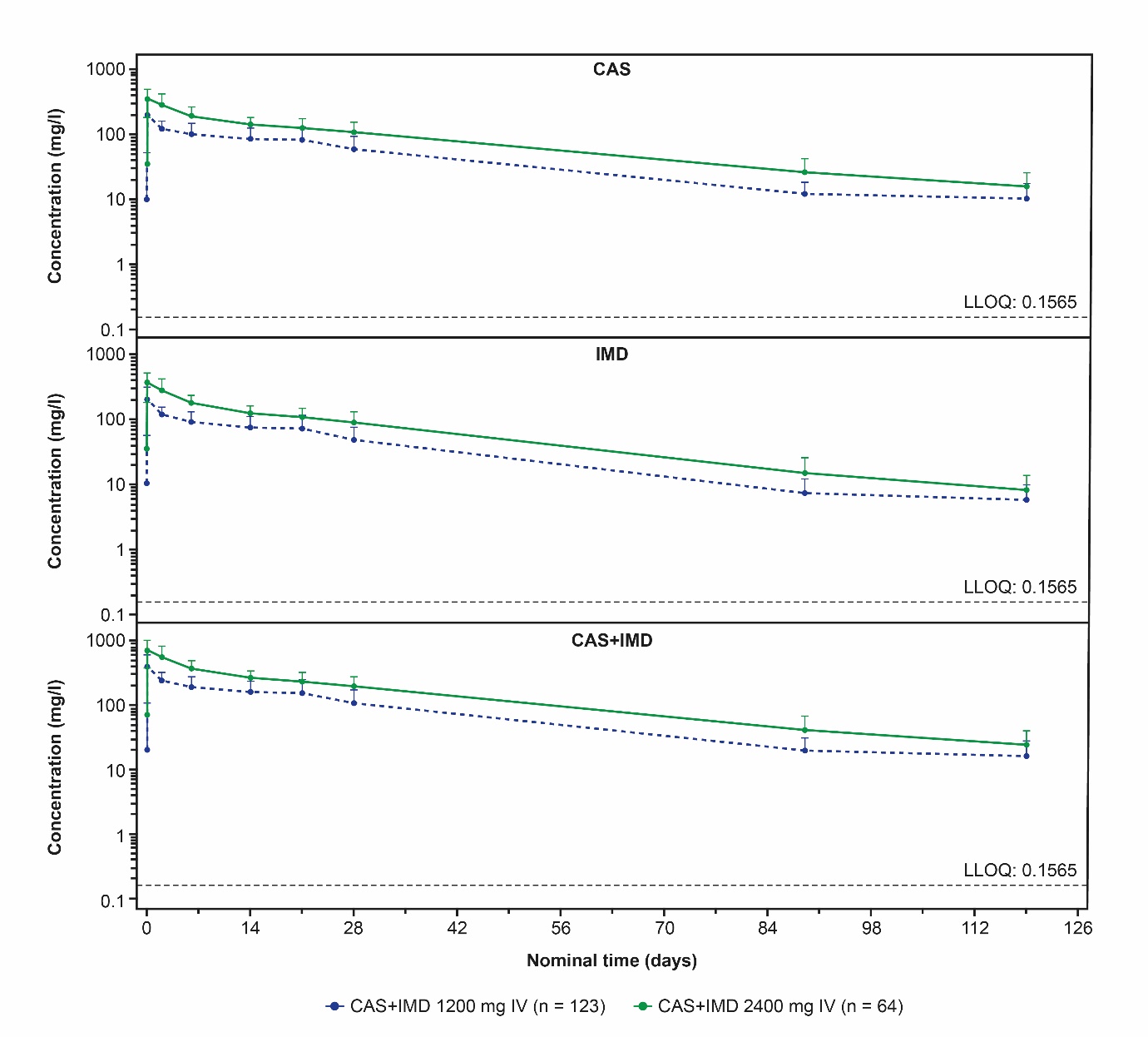


Note: Concentrations below the LLOQ were set to LLOQ/2. Participants <18 years of age were administered the body weight equivalent of CAS+IMD adult doses.

CAS, casirivimab; IMD, imdevimab; IV, intravenous; LLOQ, lower limit of quantitation; SD, standard deviation.

Supplementary Figure 6. Scatter plot of (A) TWA change from baseline, and (B) change from baseline in viral load (log_10_ copies/ml) from day 1 through day 7 versus log-scaled C_28_ of total CAS+IMD in serum by baseline viral load category in pediatric patients (CR-seronegative mFAS)

**A**


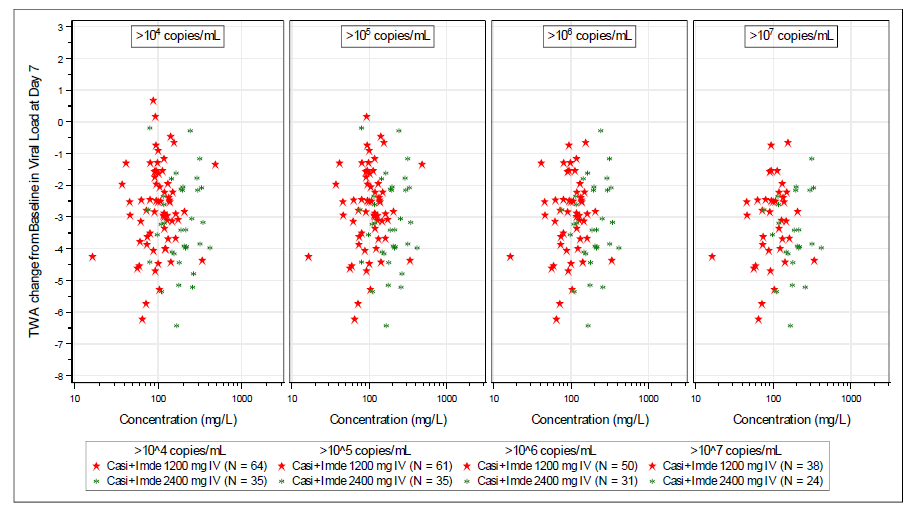


**B**


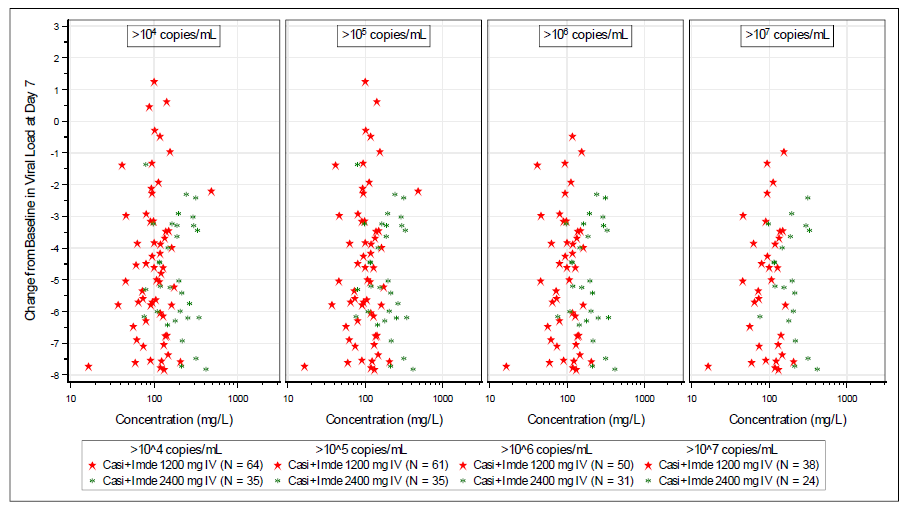


Note: Concentrations below the LLOQ were set to LLOQ/2. Participants <18 years of age were administered the body weight equivalent of CAS+IMD adult doses.

C_28_, concentration at day 28; CAS, casirivimab; CR-Seronegative mFAS, Concentration-Response analysis set for the seronegative population; IMD, imdevimab; IV, intravenous; LLOQ, lower limit of quantification; mFAS, modified full analysis set; TWA, time-weighted average.

# Reference

1. Irvin SC, Ganguly S, Weiss R, et al. REGEN-COV® antibody cocktail bioanalytical strategy: comparison of LC-MRM-MS and immunoassay methods for drug quantification. Bioanalysis. **2021**;13:1827-36.
